# Determination of 16 Hydroxyanthracene Derivatives in Food Supplements Using LC-MS/MS: Method Development and Application

**DOI:** 10.3390/toxins16120505

**Published:** 2024-11-23

**Authors:** Svetlana V. Malysheva, Benoît Guillaume, Céline Vanhee, Julien Masquelier

**Affiliations:** 1 Toxins, Organic Contaminants and Additives, Physical and Chemical Health Risks, Sciensano, Leuvensesteenweg 17, 3080 Tervuren, Belgium; benoit.guillaume@sciensano.be (B.G.); julien.masquelier@sciensano.be (J.M.); 2 Medicines and Health Products, Physical and Chemical Health Risks, Sciensano, Juliette Wytsmanstraat 14, 1050 Brussels, Belgium; celine.vanhee@sciensano.be

**Keywords:** hydroxyanthracene derivatives, aloe-emodin, emodin, sennosides, LC-MS/MS, food supplements, validation, occurrence

## Abstract

Hydroxyanthracene derivatives (HADs) are plant substances produced by a variety of plant species, including different *Aloe*, *Rheum*, and *Rhamnus* species and *Cassia senna.* These plants are often used in food supplements to improve bowel function. However, recently, the European Commission prohibited a number of HADs due to toxicological concerns. These HADs included aloin (aloin A and aloin B), aloe-emodin, emodin, and danthron. Most of the currently available analytical methods are restricted to the analysis of only these compounds and do not include other HADs. In this view, a multi-analyte method could be useful for both regulatory analysis and dietary intake studies. To this end, such a method, employing liquid chromatography–tandem mass spectrometry and targeting 16 different HADs, was developed and validated in this study. Limits of quantification were in the range from 0.025 mg kg^−1^ to 1 mg kg^−1^. The recovery of the method was within the acceptable range of 80% to 120%, with the exception of physcion. Repeatability varied from 0.5% to 11.6%, and the range for within-laboratory reproducibility was from 3.4% to 16.3%. The expanded measurement uncertainty was below 50% for all HADs. Subsequently, 24 commercial samples of food supplements and herbal infusions sourced in Belgium were analyzed. The results indicated that although the industry put a great effort into minimizing the amount of aloin and danthron present in food supplements, more than half of the products still exceeded the maximum tolerated levels suggested for aloe-emodin and emodin.

## 1. Introduction

Hydroxyanthracene derivatives (HADs) are a class of compounds naturally present in numerous plant species belonging to different botanical families and genera, among which are *Aloe*, *Rheum*, *Senna*, *Cassia*, *Frangula*, and *Rhamnus* spp. HADs are considered important biologically active components exerting a range of biological activities, including their recognized effect on the gastrointestinal system. Herbal products containing HADs are mostly appreciated for their laxative effects. According to a recent study, 40% of the worldwide population suffers from chronic gastrointestinal problems, among which 12% from chronic constipation [1].

In 2013, the European Food Safety Authority (EFSA) concluded that HADs in food could improve bowel function, but at the same time, warned that long-term use of these products and consumption at high doses may lead to potential electrolyte imbalance, impaired bowel function and dependence on laxatives [2]. In recent years, more concerns have been raised regarding the safety of HAD-containing products. Based on available scientific data, in 2018, EFSA concluded that certain HADs are genotoxic and may pose an increased risk for colorectal cancer [3]. Eventually, in 2021, aloe-emodin (ALE), emodin (EMO), the structurally related substance danthron (DAN), and aloe preparations were banned by the European Commission (EC), while preparations from certain *Rheum* sp., *Cassia senna* L. and *Rhamnus* sp. containing HADs were put under scrutiny [4]. In 2024, the EFSA Panel on Nutrition, Novel Foods and Food Allergens concluded that preparations with rhubarb, frangula, cascara, and senna pose safety concerns for genotoxicity [5].

Consequently, the industry introduced filtration/purification processes aiming to remove HADs, although these methodologies are not completely efficient. Nevertheless, in order to guarantee the safety of foodstuffs on the market, analytical methods for HADs were implemented. Several literature reports exist describing methods for quantification of ALE, EMO, aloin A (ALA), aloin B (ALB), and some other HADs in botanical preparations [6]. Not all these methods have been validated, or only partial validation was performed, or they focused solely on the HADs mentioned above. A great part of the methods made use of the high-performance liquid chromatography (HPLC) technique with ultraviolet (UV) or diode-array detectors (DAD) [7,8,9,10,11,12]. Kline et al. developed and validated a quantitative HPLC-UV method for the analysis of ALA, ALB, and ALE in *Aloe vera* raw materials and finished products [7]. Using the same technique, Yılmaz and Kolak quantified RHE, EMO, CHR, and PHY in *Rhamnus petiolaris* fruit extracts [8], while Al-Hamoud et al. analyzed CHR in *Senna occidentalis* extract [9]. The latter study made use of ultrasound-assisted extraction, which was optimized using response surface methodology. ALE in *Aloe vera* extracts and commercial formulations was determined by Mandrioli et al. using HPLC with tandem UV absorption and fluorescence detection (FD) [10]. The authors emphasized that coupling UV and FD allowed comparison of the data obtained with both detection methods, elevating the level of confidence in the results. A rapid method utilizing UHPLC coupled to a photodiode array detector (PDA) was developed for the quantification of five anthraquinones (ALE, EMO, PHY, RHE, and CHR) in *Polygonum multiflorum* [13]. Ultrasonic extraction followed by dispersive solid-phase extraction (dSPE) were used for the preparation of the samples. This method aided in demonstrating large differences in the HAD content in *Polygonum multiflorum* samples from different regions. Liquid (LC) or gas chromatography (GC) combined with mass spectrometry (MS) was also used for the analysis of HADs in botanical preparations [14,15,16,17]. Loschi et al. developed an LC-DAD-MS method for the analysis of five HADs, namely ALA, ALB, ALE, EMO, and DAN, in food supplements and plant materials [11]. A GC-MS method was developed by Dai et al. for the determination of four anthraquinones, ALE, EMO, CHR and PHY, in rhubarb, the traditional Chinese medicinal herb [14]. Osthole was used as an internal standard, and the MS detection was accomplished in selected ion monitoring mode to increase the sensitivity. Recently, a UHLC-MS/MS quantification of HADs in *Aloe vera* (L.) Burm. f. gel commercial beverages was presented by Di Minno et al. [18]. The method targeted only ALA, ALB, EMO, and ALE and was characterized by a fast separation of the analytes, which was achieved within 5 min. Another analytical method was validated for the simultaneous quantification of ALE, EMO, PHY, RHE, and CHR among two other physiologically relevant active components and eight adulterants [16]. As an analytical technique, HPLC-MS/MS was optimized, and the target matrices included slimming foods and herbal products. The heteronuclear multiple quantum correlation spectroscopy technique was used for the determination of the total HAD content in plant materials [19], while a two-dimensional quantitative nuclear magnetic resonance was utilized to determine total sennosides and separately individual content of SENA, SENA1, and SENB in senna leaflets, pods and tablets [20]. The method performance was satisfactory, except for precision for SENA1 and SENB, which was mainly attributed to the low resolution of the heteronuclear single quantum correlation spectroscopy signals for these HADs. Overall, the main drawbacks of the published methods are either a lack of specificity and/or sensitivity or a limited number of HADs included. Having diverse HADs in the method is pivotal to having a more complete picture of the amount and type of HADs present in the samples. Indeed, a previous study performed by Fierens and Corthout [21] on food supplements retrieved from the Belgian market showed large variability in total HAD content but lacked data on individual HADs. Consequently, the estimation of the exposure to HADs from food supplements based on the recommended daily doses of these products was only possible for a limited number of derivatives due to this lack of data for the other HADs [3].

The objective of this work was to develop and validate an analytical method based on ultra-high-performance liquid chromatography–tandem mass spectrometry (UHPLC-MS/MS) for the detection and quantification of individual HADs in a single run. The advantages of this technique include high sensitivity, specificity, and the possibility to analyze many analytes simultaneously. The target compounds included ALA, ALB, ALE, chrysophanol (CHR), DAN, EMO, frangulin A (FRA), frangulin B (FRB), glucofrangulin A (GFA), glucofrangulin B (GFB), physcion (PHY), rhein (RHE), sennoside A (SENA), sennoside A1 (SENA1), sennoside B (SENB), and sennoside C (SENC).

## 2. Results and Discussion

### 2.1. Optimization of LC-MS/MS Conditions

For the optimization of the MS signal of HADs, mobile phases with methanol (MeOH) and acetonitrile (ACN) as organic modifiers and with formic acid (HCOOH), acetic acid (CH_3_COOH), ammonium formate (HCOONH_4_), and ammonium bicarbonate (NH_4_HCO_3_) as additives were tested (Supplementary Figure S1). Mobile phases with no additives were also investigated. The spectra were recorded in positive (ESI(+)) and negative (ESI(−)) electrospray ionization modes using flow injection analysis. It was apparent that, overall, MeOH with or without additives generated a higher MS signal compared to ACN. The mobile phases without an additive generated poor signal for DAN, CHR, and ALE. Among the tested additives, HCOOH at 0.1% provided a better signal for the majority of HADs. A few exceptions were FRB and GFA showing a better signal in the mobile phase with 10 mM NH_4_HCO_3_ (MeOH as an organic modifier) and ALE demonstrating a higher signal in the mobile phase with 0.1% HCOOH and ACN as an organic modifier. As a compromise, the mobile phase with MeOH and 0.1% HCOOH was selected for further optimization.

Regarding the selection of ionization mode, aloins, frangulins, ALE, CHR, DAN, and PHY were better ionized in ESI(+), generating abundant [M+H]^+^ precursor ions (Table 1). A sufficiently intense signal in ESI(+) was also obtained for GFB, SENA, and SENA1 through the formation of [M+Na]^+^ and [M+NH_4_]^+^ (the latter was only for sennosides) adducts. Knowing that sodium adducts are generally less robust, ESI(−) was preferred for the analysis of these three HADs, as they also formed abundant deprotonated molecules. ESI(−) was also chosen for the detection of EMO, GFA, RHE, SENB, and SENC. Notably, the most abundant signal for both glucofrangulins was obtained using formic acid adducts (Table 1). SENB and SENC were well ionized in both ESI(+) and ESI(−) modes, but somewhat better sensitivity was achieved in ESI(−). In the final method, for higher specificity and sensitivity, the data were acquired in multiple reaction monitoring (MRM) mode. Table 1 summarizes the optimized precursor and product ions for the MS/MS detection of HADs.

**Figure 1:**
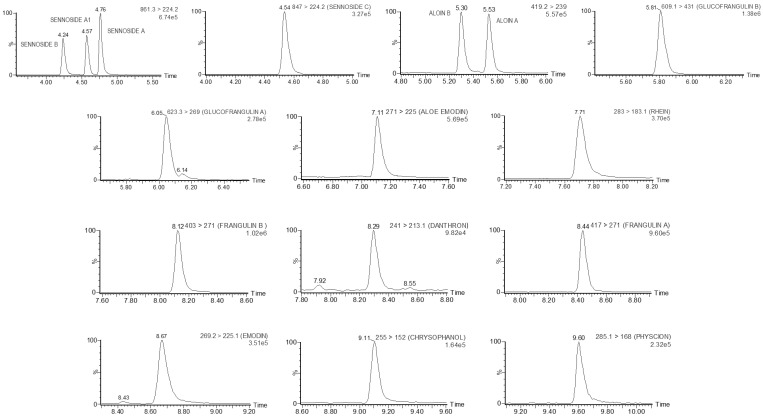
LC-MS/MS multiple reaction monitoring (MRM) chromatograms of a blank food supplement fortified with 16 HADs at the limit of quantification (LOQ) level. The LOQ levels for each HAD are specified in Table 2. For each analyte, the most abundant MRM transition is displayed. The vertical axes represent relative peak intensity (normalized to 100%), while the horizontal axes display retention time (in min). The chromatographic conditions used were as described in Section 4.4.

LC columns, previously used in multi-target analysis of some HADs and other natural toxins [13,22,23,24,25,26], namely ACQUITY^TM^ UPLC^TM^ BEH C18 (1.7 µm, 2.1 × 100 mm), ACQUITY UPLC HSS T3 (1.8 µm, 2.1 × 100 mm), and ACQUITY UPLC BEH Phenyl (1.7 µm, 2.1 × 100 mm), were tested for the separation of HADs using the optimal mobile phase, and similar satisfactory results were obtained. Finally, the first-listed column was chosen for the method, and the gradient program was described in Section 4.4. Figure 1 displays the separation profile of HADs under the optimized LC conditions, while information on the retention time is summarized in Table 1. Importantly, the peaks of HADs with the same target precursor and product ions, namely, sennosides SENA, SENA1, and SENB, and aloins ALA and ALB were baseline separated.

### 2.2. Optimization of Sample Preparation

The sample preparation procedure was optimized using fine powder of a multi-component plant-based food supplement intended to support intestinal function.

Different organic solvents and/or their combinations were tested for the extraction of HADs from the plant-based material and included MeOH, ACN, ethanol (EtOH), and acetone (ACE) (Supplementary Figure S2). EtOH or ACE with 30% H_2_O, commonly used in the extraction of anthraquinones [13,27,28], demonstrated satisfactory results with regard to extraction efficiency. In the case of EtOH, the addition of H_2_O greatly improved the recovery of aloins, ALE, glucofrangulins, and sennosides. Nonetheless, the estimated limits of quantification (LOQs) for SENA and PHY using EtOH/H_2_O (70/30, *v/v*) for the extraction were considered rather high. ACN alone or in combination with H_2_O in different proportions showed lower extraction efficiency for ALB and sennosides.

The use of MeOH as an extraction solvent resulted in a somewhat lower yield for SENA, SENA1, and SENB. The addition of 1% CH_3_COOH only slightly improved the recovery of these sennosides, while the use of MeOH in combination with 1% HCOOH provided good results for all HADs (extraction efficiency > 70%). With this latter extraction mixture, good estimated LOQ values were obtained; however, the downside of this protocol was the distorted LC peak shape for sennosides due to the higher elution strength of the sample solvent compared to the mobile phase. The peaks of the other HADs were not affected.

Another protocol, with the addition of H_2_O to the extraction solvent MeOH, was also tested. Surely, the higher percentage of H_2_O led to a better peak shape for sennosides; however, at 50% H_2_O in the solvent mixture, the recovery of PHY, DAN, CHR, and EMO reduced down to about 20–45%. The optimal combination in terms of extraction efficiency and sensitivity was found to be 20% H_2_O in MeOH.

Different sample-to-solvent ratios (between 1:7.5 (*w/v*) to 1:15 (*w/v*)) were investigated, and the final selection was the ratio of 1:15 (*w/v*), providing good sensitivity.

Furthermore, different clean-up methods with or without subsequent extract evaporation were tested in order to improve the method sensitivity even further and/or allow for the solvent switch before injection into the LC system for better peak shape. None of the target HADs were detected after the application of solid-phase extraction (SPE) on the graphitized carbon cartridges (Supelclean ENVI-Carb). Low to no recovery of the analytes was observed in the case of dSPE based on C18 and/or primary secondary amine (PSA) sorbents. SPE performed on the Oasis HLB cartridges demonstrated low efficiency for EMO, DAN, CHR, and PHY using MeOH for the elution. Acidification of the elution solvent with 1% HCOOH resulted in improved efficiency for the problematic HADs but still reached only about 30% for PHY and required an increased elution volume (3 × 3 mL). Similar results were obtained for the Discovery DSC-18 cartridges. In the end, considering the unsatisfactory efficiency for some HADs and the lack of clear benefit compared to the protocol with only extraction, the purification of sample extracts was dismissed.

The final sample preparation procedure for food supplements consisted of a single-step solid-liquid extraction with MeOH/H_2_O (80/20, *v/v*), subsequent extract dilution with H_2_O to reach its volume concentration of 50% in the extract, and filtration (see Section 4.3). The dilution was necessary to resolve the issue of distorted LC peak shape for sennosides.

### 2.3. Method Validation

The method validation data for the analysis of HADs in food supplements are summarized in Table 2 and Table 3.

The selectivity of the method was assessed by analyzing different blank plant-based matrices. No interfering peaks in the region of the analyte elution, which necessitated further improvement of the LC separation, MS detection, or sample preparation procedure, were present (Supplementary Figure S3). The matrix effect ranged from 32% to 114%. It was revealed that all HADs, with the exception of DAN, had high to moderate signal suppression. The signal of ALB and GFA was affected by the matrix the most (Table 2). The utilization of the isotopically labeled internal standards (IS), namely aloe-emodin-*d_4_* (ALE-D4), danthron-*d*_4_ (DAN-D4), emodin-*d*_4_ (EMO-D4), and physcion-*d*_3_ (PHY-D3), in the control of matrix effects was evaluated. Such analogs were not commercially available for each of the target HADs. While for a number of HADs the matrix effects could be improved, for sennosides and glucofrangulins, none of the IS was suitable. Initially, for PHY, its analog PHY-D3 was used, but due to inconsistent day-to-day method validation results using this IS, it was eventually excluded. This could be attributed to the position of deuterium in the structure and its stability. Unlike ALE-D4, DAN-D4, and EMO-D4, PHY-D3 produced no MS fragments with the labeled site on the portion of the molecule that is being quantitated. The final choice of the IS is given in Table 2.

Matrix-matched calibration was used for quantification, and the starting calibration point for all HADs corresponded to the respective LOQ level. Mandel’s test showed that the linear regression model was the best fit, and the coefficients of determination were all above 0.99. The linear range is specified in Table 2. The LOQs varied from 0.025 mg kg^−1^ to 1 mg kg^−1^, with EMO being the most sensitive compound and GFB and SENA demonstrating the lowest sensitivity (Table 2). Compared to other studies, the current method demonstrated much lower LOQs for sennosides, aloins, frangulins, ALE, EMO, CHR, PHY, DAN, and RHE [11,12,13,26,29,30,31,32,33,34]. While Loschi et al. achieved concentrations for aloins, ALE, EMO, and DAN only as low as between 42 ng mL^−1^ and 94 ng mL^−1^ [11], the current study presented LOQs in the range from 1.04 ng mL^−1^ to 8.33 ng mL^−1^. In a previous report, frangulins were quantified at the lowest concentration of 61.4 ng mL^−1^ [26], which was much higher than the LOQ of 4.17 ng mL^−1^ obtained in the current study. However, Fung et al. obtained a more sensitive detection for SENA, SENB, and ALE at 4.73 ng mL^−1^, 4.32 ng mL^−1^, and 1.51 ng mL^−1^, respectively, in comparison to 41.67 ng mL^−1^, 31.25 ng mL^−1^, and 8.33 ng mL^−1^, respectively, achieved in the current study [22]. Concurrently, Fung et al. obtained much lower sensitivity for PHY (10.29 ng mL^−1^), CHR (8.44 ng mL^−1^), and EMO (2.08 ng mL^−1^). LOQs for these HADs in the current method were as low as 4.17 ng mL^−1^, 4.17 ng mL^−1^, and 1.04 ng mL^−1^, respectively. Compared to the data of Wu et al. [32], the method described in the current study achieved approximately three to five times lower LOQs for SENA, RHE, and ALE. LOQ values for GFA and GFB were not reported in previous studies. Overall, the LOQs obtained in the present study were considerably lower than the threshold of 1 ppm proposed by the EC for reliable quantification of ALE, EMO, and the sum of ALA and ALB [35].

The method was validated at three concentration levels, including the LOQ level, and the recoveries ranged from 80.1% (CHR) to 119.3% (GFB) (Table 3) and thus were within the acceptance limit of 80%–120% [36]. PHY was the only exception, with a mean recovery of 50.7%; for this reason, a recovery correction was implemented for the quantification of this HAD. Previously published studies reported recovery of HADs in the range from 71.5% to 118.4% [13,14,16,18,29,30]. Although the recovery value for PHY in the current research was below the desired limit, it was amply repeatable (mean: 2.3%) and reproducible (mean: 12.4%), eventually allowing for reliable recovery correction in the quantification of samples. Further optimization of extraction and/or clean-up procedures with the purpose of improving the recovery of PHY proved to be unrewarding due to the fact that the method covered analytes with different physico-chemical properties. The final sample preparation protocol was a compromise for having all target HADs analyzed using an easy-to-apply protocol and with good sensitivity.

The range for repeatability was from 0.5% (SENA) to 11.6% (DAN), while within-laboratory reproducibility varied from 3.6% (SENA) to 16.3% (DAN) (Table 3). Commission Implementing Regulation (EU) 2021/808 provides acceptance criteria for within-laboratory reproducibility that correspond to the validated concentration level [36]. This parameter was much lower than the specified acceptance limit for all HADs and for all concentration levels (Table 3). The expanded measurement uncertainty (MU) ranged from 12.2% (GFA) to 49.2% (DAN) (Table 3). It has to be noted that the uncertainty of bias was not included in the calculation of expanded MU for PHY due to the application of recovery correction in quantification. Overall, the method was found to be fit for purpose. It is important to mention that this validated method covers a range of HADs beyond two to five usual HADs, which were targeted by other methods, thus providing a more comprehensive assessment of HAD content of plant-based samples. The developed method was subsequently applied to the analysis of HADs in commercial products.

**Table 3:** Recovery, repeatability (relative standard deviation (RSD_r_)), reproducibility (RSD_RW_), and expanded measurement uncertainty (MU) for the analysis of HADs in food supplements.

HAD	Concentration Level (mg kg^−1^)	Recovery (%)	RSD_r_ (%)	RSD_RW_ (%)	Acceptance Criteria for RSD_RW_ (%) [36]	Expanded MU (%)
Aloin A (ALA)	0.05	101.1	7.1	13.4	25	37.2
	0.15	108.5	4.9	6.3	22	24.8
	0.25	99.5	2.6	7.3	22	20.1
Aloin B (ALB)	0.1	104.5	6.5	15.1	25	43.4
	0.3	112.1	5.0	5.0	22	28.2
	0.5	102.9	2.3	4.5	22	13.8
Aloe-emodin (ALE)	0.2	98.6	7.2	14.3	22	39.2
	0.6	103.7	4.5	6.0	22	18.3
	1.0	96.3	2.2	5.5	22	16.7
Chrysophanol (CHR)	0.1	80.1	7.5	11.1	25	48.5
	0.3	88.9	7.3	7.8	22	30.0
	0.5	81.5	1.2	5.1	22	39.1
Danthron (DAN)	0.2	87.5	11.6	16.3	22	49.2
	0.6	96.9	5.9	9.0	22	25.1
	1.0	92.7	4.0	7.8	22	25.3
Emodin (EMO)	0.025	83.8	8.3	14.1	25	48.4
	0.075	94.0	8.0	15.3	25	42.5
	0.125	87.5	3.4	13.5	22	42.9
Frangulin A (FRA)	0.1	84.0	9.1	14.3	25	48.6
	0.3	103.7	4.9	5.4	22	16.9
	0.5	95.3	2.5	4.4	22	15.0
Frangulin B (FRB)	0.1	81.1	8.8	11.2	25	47.1
	0.3	99.8	4.9	6.8	22	18.7
	0.5	92.0	2.7	5.5	22	21.6
Glucofrangulin A (GFA)	0.1	105.9	1.6	13.3	25	39.3
	0.3	106.3	1.5	5.4	22	19.7
	0.5	101.3	1.3	4.3	22	12.2
Glucofrangulin B (GFB)	1.0	108.7	1.9	14.3	22	44.5
	3.0	119.3	1.1	5.4	16	41.9
	5.0	116.3	0.8	4.4	16	35.0
Physcion (PHY) ^1^	0.1	49.2	3.0	8.3	25	17.5
	0.3	50.9	1.6	13.4	22	28.2
	0.5	52.0	2.4	15.4	22	32.5
Rhein (RHE)	0.1	103.9	6.1	6.4	25	19.7
	0.3	107.4	4.2	8.3	22	27.8
	0.5	100.7	2.3	7.0	22	19.4
Sennoside A (SENA)	1.0	87.9	0.6	13.4	22	42.4
	3.0	96.0	1.2	5.9	16	17.8
	5.0	93.4	0.9	3.6	16	16.1
Sennoside A1 (SENA1)	0.75	95.9	1.5	6.0	22	18.1
	2.25	93.4	0.6	7.4	16	23.7
	3.75	91.0	0.5	7.2	16	26.3
Sennoside B (SENB)	0.75	93.5	2.1	6.1	22	20.8
	2.25	93.9	1.2	4.7	16	17.5
	3.75	92.5	0.7	5.0	16	20.0
Sennoside C (SENC)	0.5	100.5	1.7	13.8	22	38.0
	1.5	107.2	1.0	9.0	16	29.3
	2.5	103.1	0.8	8.4	16	24.3

^1^ The recovery was calculated using the uncorrected measured concentration in order to demonstrate that for the quantification recovery correction was necessary. The uncertainty of bias was not included in the calculation of the expanded MU.

### 2.4. Method Application

**Figure 2:**
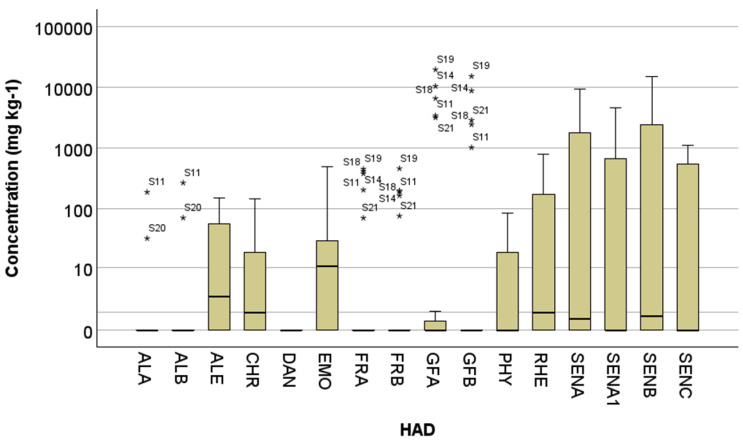
Distribution of HAD content in commercial food supplements and herbal infusions. The bottom and top of the box correspond to the lower quartile (25th percentile) and the upper quartile (75th percentile), respectively. The line inside the box represents the median (50th percentile). The ends of the whiskers correspond to the lowest and highest observations that are still within 1.5-fold of the interquartile range (corresponding to the length of the box). Observations beyond the ends of the whiskers are marked with an asterisk and labeled with a sample code. The spacings between the different parts of the box give an indication of the degree of spread and skewness of the data. Given the large distribution in HAD concentrations, a logarithmic scale is applied to the y-axis for better visualization.

The validated LC-MS/MS method was utilized for the analysis of 24 commercial samples of food supplements and dry herbal infusions purchased on the Belgian market. Most of the acquired samples contained senna extracts and rhubarb and were intended to support bowel function. Supplementary Table S1 provides detailed information on the samples’ ingredients, while Figure 2 and Supplementary Table S2 summarize the measured concentrations of HADs in these products.

The target HADs in the samples were identified in accordance with the requirements of Commission Implementing Regulation (EU) 2021/808 [36]. This included correspondence of the retention time and ion ratio of the analyte in the sample to that of the matrix-matched standards with a tolerance of 0.1 min and a relative deviation of 40%, respectively. In case an internal standard is used, a maximum deviation for the relative retention time of the analyte in the sample is set to 1%. For the analysis and data processing, the most abundant product ion was used for quantification, while the second ion was used for confirmation of the detected HADs (see Table 1). For samples with concentrations exceeding the working range of the method, a re-analysis was performed by diluting the sample extract with a blank matrix extract used for the preparation of the matrix-matched calibration curve. The dilution factor was selected using the estimated concentration from the initial analysis with the intention of obtaining a response within the working range.

In 7 out of 24 samples, no HADs were detected above the LOQ level. Among the different analytes, only DAN was not found in the samples, which can be explained by the synthetic origin of this HAD. The highest number of HADs present in the same sample was equal to 13 in a food supplement containing buckthorn, rhubarb, and senna and in a herbal infusion with senna. Eleven HADs simultaneously occurred in food supplements containing *Frangula alnus*, *Aloe vera*, and senna. Ten HADs were found in a food supplement containing, among others, senna leaves and rhubarb. The level of 1 ppm was exceeded in 14 out of 24 analyzed samples (58%) for EMO, 12 samples (50%) for ALE and 2 samples (8%) for the sum of ALA and ALB.

The most frequently detected HAD was EMO (67% of samples), followed by CHR (58%) and RHE (54%). ALE, SENA, and SENB were measured in 50% of the samples, while the least detected HADs were ALA (8%) and ALB (8%). The individual concentrations of HADs in positive samples ranged from 0.13 mg kg^−1^ to 19.81 g kg^−1^. The highest levels were found for GFA (19.81 g kg^−1^) and GFB (15.39 g kg^−1^) in a food supplement based on dirt tree bark, SENB (15.39 g kg^−1^) in a food supplement containing, among others, senna pods and rhubarb root extract, and GFA (10.55 g kg^−1^) in a herbal infusion of *Rhamnus frangula*. As regards the sample with senna pods and rhubarb extract, on the package, it is stated that the product contains 18 mg sennosides per tablet of 345 mg, which corresponds to 52.2 g kg^−1^ of total sennosides. The current analysis demonstrated that this product contained 30.2 g kg^−1^ of sennosides, the sum of SENA, SENA1, SENB, and SENC, implying that other isomers not covered by the current method might be present [20]. Notably, this sample also showed the highest total concentration of HADs in this work (sum of HADs: 41.05 g kg^−1^). Other highly contaminated samples were a food supplement with dirk tree bark (sum of HADs: 36.69 g kg^−1^) and *Rhamnus frangula* herbal infusion (20.09 g kg^−1^).

It is important to mention that, with the exception of a few samples with low HAD concentrations, if one sennoside was detected in a sample, the other targeted sennosides were also present. This observation was also valid for ALA with ALB, and FRA with FRB, GFA and GFB.

The results of this small-scale market study undoubtedly demonstrate the importance of putting in place analytical methods for HADs in food supplements and other plant-based matrices. In vitro genotoxicity testing suggested that HADs, particularly ALE, interact with mammalian DNA under certain conditions. Based on different toxicological studies, EFSA concluded that HADs should be considered genotoxic and carcinogenic unless there are specific data to the contrary, such as for RHE [3]. Although recent in vivo studies on dried *Aloe ferox* juice and ALE indicated against DNA damage [37,38], the conclusion of EFSA on the safety of HADs remained unchanged [5]. At present, no daily intake of HADs that does not give rise to health concerns can be established. Given that about half of the commercial products in the current study exceeded the tolerated limit for ALE and EMO, this occurrence data certainly require more attention, as they highlight a health concern for the consumers [35]. Additionally, it should not be overlooked that two products contained 13 out of 16 target HADs simultaneously, highlighting the necessity of performing combined dietary exposure assessment to guarantee a high level of public health protection.

## 3. Conclusions

In this study, a UHPLC-MS/MS method for the simultaneous quantification of 16 HADs in food supplements was developed and validated. The validation study showed satisfactory performance parameters and good sensitivity of the method. Analysis of commercial food supplements and herbal infusions revealed the presence of sennosides, aloins, frangulins, glucofrangulins, and other target HADs, except for DAN. About 60% of the samples significantly exceeded the threshold for the content of a number of HADs proposed by the EC, thus stressing the importance of this method and the collected occurrence data from a controlling and regulatory point of view. The presented method is of critical importance for the scientific community and industry since the recent prohibition of certain HADs in preparations. The method can also be used as a tool in collecting more occurrence data for a wider range of HADs in plant-based products and facilitating a more accurate dietary exposure assessment.

## 4. Materials and Methods

### 4.1. Reagents and Standards

MeOH Optima^TM^ LC/MS from Fisher Scientific (Merelbeke, Belgium) was used. HCOONH_4_ ULC/MS, HCOOH ULC/MS, CH_3_COOH ULC/MS, ACN ULC/MS, ACE, and 2-propanol LC-MS were supplied by Biosolve BV (Valkenswaard, The Netherlands). Dimethyl sulfoxide (DMSO) and NH_4_HCO_3_ LC-MS LiChropur™ were obtained from Merck (Zwijndrecht, The Netherlands), while EtOH was purchased from VWR International (Leuven, Belgium). Waters (Antwerp, Belgium) was the supplier of Oasis^®^ HLB Vac cartridges (3 cc, 60 mg). H_2_O was purified using a Milli-Q purification system (Millipore Corp., Bedford, MA, USA). Phenex-RC syringe filters (15 mm, 0.2 µm) were acquired from Phenomenex (Utrecht, The Netherlands). Single-use 3 mL syringes HENKE-JECT^®^, and PP 50 mL centrifuge tubes were supplied by VWR International. Supelclean^TM^ ENVI-Carb^TM^ (3 mL, 250 mg) SPE tubes and Discovery^®^ DSC-18 (6 mL, 500 mg) tubes were acquired from Merck. Bondesil C18 (40 μm) and Bondesil PSA (40 µm) bulk sorbents were obtained from Agilent (Diegem, Belgium).

Reference standards of the native HADs were purchased from PhytoLab GmbH &Co.KG (Vestenbergsgreuth, Germany), while the labeled analogs were supplied by LGC Standards (Molsheim, France). ALE, CHR, RHE, SENA, SENA1, and SENC were dissolved in DMSO at 1 mg mL^−1^. ALA, ALB, GFA, and EMO-D4 were dissolved in MeOH at 1 mg mL^−1^. EtOH was used to dissolve DAN and DAN-D4 at 0.5 mg mL^−1^. FRA, FRB, and PHY-D3 were dissolved at 0.5 mg mL^−1^ in DMSO/2-propanol (50/50, *v/v*), MeOH/DMSO (90/10, *v/v*), and DMSO, respectively. The stock solutions of PHY and SENB were prepared at 0.4 mg mL^−1^ in DMSO and MeOH, respectively. EMO was dissolved in MeOH at 0.25 mg mL^−1^, while ALE-D4 was prepared in MeOH at 0.1 mg mL^−1^. GFB was dissolved in MeOH at 0.04 mg mL^−1^. The intermediate and working solutions were prepared in MeOH. All solutions of HADs and IS were stored at −20°C.

### 4.2. Samples

The majority of products for the analysis of HADs were purchased in local health food shops and supermarkets, while others were obtained from online pharmacies. A detailed overview of the samples is given in Supplementary Table S1. Prior to extraction, samples were finely ground and/or homogenized.

### 4.3. Sample Preparation

Two grams of a food supplement were weighed in a 50 mL PP extraction tube. Thirty mL of MeOH/H_2_O (80/20, *v/v*) was added, and the sample was extracted for 45 min on an overhead shaker (Heidolph Reax 2, Fisher Scientific). After centrifugation for 15 min at 3181× *g*, 900 µL of the supernatant was combined with 540 µL H_2_O. Prior to injection into the LC-MS/MS system, the diluted extract was filtered through 0.2 µm Phenex-RC filter using a syringe and spiked with a mixture of IS at levels corresponding to 1.79 mg kg^−1^ for ALE-D4, 1.19 mg kg^−1^ for DAN-D4 and 0.07 mg kg^−1^ for EMO-D4.

### 4.4. LC-MS/MS Conditions

Analysis of HADs involved separation on an ACQUITY UPLC H-class system (Waters, Milford, MA, USA) equipped with an ACQUITY UPLC BEH C18 column (1.7 µm, 2.1 × 100 mm; Waters, Antwerp, Belgium) and an ACQUITY UPLC BEH C18 guard column (1.7 µm, 2.1 × 5 mm; Waters, Antwerp, Belgium). The column temperature was kept at 60 °C. The mobile phase used was (A) H_2_O containing 0.1% HCOOH and (B) MeOH containing 0.1% HCOOH at a flow rate of 0.4 mL min^−1^. The gradient program started with 5% B, linearly increasing to 85% B in 9 min. This mobile phase ratio was kept for 1.5 min and changed to 5% B within 0.5 min. Finally, the column was equilibrated with 5% B for 3 min. The total run time was 15 min, and the injection volume was 10 µL.

After separation, the analytes were detected by a Xevo TQ-S triple quadrupole mass spectrometer (Waters, Milford, MA, USA) operated in ESI(+) and ESI(−) modes. The MS parameters for both ionization modes were as follows: capillary voltage: 1.00 kV, source temperature: 150 °C, desolvation temperature: 450 °C, cone gas flow: 150 L h^−1^, desolvation gas flow: 1000 L h^−1^, collision gas flow: 0.15 mL min^−1^, source offset: 30 V. The data were acquired in MRM mode, and the optimized MRM parameters are given in Table 1.

### 4.5. Validation

Method validation was performed on a multi-component fiber-based food supplement for supporting intestinal function, and the following parameters were determined: matrix effects, linearity, sensitivity, recovery, repeatability, within-laboratory reproducibility, and measurement uncertainty.

For the evaluation of matrix effects, matrix-matched calibration curves and curves dissolved in the solvent, i.e., MeOH/H_2_O (50:50, *v/v*), were prepared. The slopes of the curves were compared by means of a *t*-test, and the matrix effect was calculated as a relative percentage of the ratio of the curve slopes. For the linearity, matrix-matched calibration curves of at least six fortification levels were prepared and analyzed in triplicate, and Mandel’s test was used to assess the suitability of linear and quadratic regression models.

Sensitivity in terms of LOQ was defined as the lowest concentration level for which the method performance criteria were satisfied. The determination of recovery, repeatability, within-laboratory reproducibility, and measurement uncertainty was performed based on analyte-free matrix samples fortified in triplicate at three concentration levels (see Table 3). This analysis set-up was repeated on two other days by the same operator. Recovery was calculated as the relative percentage of the difference between the measured concentration and the fortified concentration. Repeatability and within-laboratory reproducibility are expressed as RSD under repeatability and reproducibility conditions, respectively. The expanded MU was calculated as a square root of the sum of squares of the uncertainties of bias and reproducibility and was multiplied by a coverage factor of 2 for 95% confidence.

## Figures and Tables

**Table 1:** Retention times and optimized multiple reaction monitoring (MRM) parameters for the UHPLC-ESI-MS/MS analysis of HADs.

HAD	Retention Time (min)	Ionization Mode	Precursor Ion (*m/z*)	Cone Voltage (V)	Product Ion (*m/z*)	Collision Energy (eV)
Aloin A (ALA)	5.53	ESI(+)	419.2 [M+H]^+^	15	239.0 ^1^	10
					211.0	25
Aloin B (ALB)	5.30	ESI(+)	419.2 [M+H]^+^	20	239.0 ^1^	10
					211.0	25
Aloe-emodin (ALE)	7.11	ESI(+)	271.0 [M+H]^+^	15	225.0 ^1^	25
					197.0	30
Chrysophanol (CHR)	9.11	ESI(+)	255.0 [M+H]^+^	50	152.0 ^1^	35
					181.0	25
Danthron (DAN)	8.29	ESI(+)	241.0 [M+H]^+^	30	213.1 ^1^	25
					167.1	25
Emodin (EMO)	8.67	ESI(−)	269.2 [M−H]^−^	30	225.1 ^1^	30
					241.1	30
Frangulin A (FRA)	8.44	ESI(+)	417.0 [M+H]^+^	15	271.0 ^1^	15
					197.0	40
Frangulin B (FRB)	8.12	ESI(+)	403.0 [M+H]^+^	20	271.0 ^1^	20
					197.0	45
Glucofrangulin A (GFA)	6.05	ESI(−)	623.3 [M+HCOOH−H]^−^	30	269.0 ^1^	60
					431.0	30
Glucofrangulin B (GFB)	5.81	ESI(−)	609.1 [M+HCOOH−H]^−^	30	431.0 ^1^	20
					269.0	30
Physcion (PHY)	9.60	ESI(+)	285.1 [M+H]^+^	30	168.0 ^1^	35
					139.2	55
Rhein (RHE)	7.71	ESI(−)	283.0 [M−H]^−^	30	183.1 ^1^	30
					211.1	30
Sennoside A (SENA)	4.76	ESI(−)	861.3 [M−H]^−^	30	224.2 ^1^	50
					386.3	50
Sennoside A1 (SENA1)	4.57	ESI(−)	861.3 [M−H]^−^	30	224.2 ^1^	50
					386.3	50
Sennoside B (SENB)	4.24	ESI(−)	861.3 [M−H]^−^	30	224.2 ^1^	50
					386.3	50
Sennoside C (SENC)	4.54	ESI(−)	847.0 [M−H]^−^	30	224.2 ^1^	50
					386.3	50
Aloe-emodin-*d*_4_ (ALE-D4)	7.09	ESI(+)	275.1 [M+H]^+^	15	229.1 ^1^	20
					245.1	20
Danthron-*d*_4_ (DAN-D4)	8.27	ESI(+)	245.1 [M+H]^+^	20	143.0 ^1^	35
					161.1	30
Emodin-*d*_4_ (EMO-D4)	8.65	ESI(−)	273.1 [M−H]^−^	30	229.0 ^1^	30
					245.1	30
Physcion-*d*_3_ (PHY-D3)	9.58	ESI(+)	288.1 [M+H]^+^	20	168.0 ^1^	40
					242.0	30

^1^ The most abundant product ion.

**Table 2:** Limit of quantification (LOQ), matrix effect, and linearity data for the analysis of HADs in food supplements.

HAD	LOQ (mg kg^−1^)	LOQ (mg L^−1^)	Matrix Effect (%)	Linear Range (mg kg^−1^)	Coefficient of Determination R^2^	Regression Model	Internal Standard Used
Aloin A (ALA)	0.05	0.002	40	0.05–0.5	0.9923	Linear	ALE-D4
Aloin B (ALB)	0.1	0.004	32	0.1–1.0	0.9925	Linear	ALE-D4
Aloe-emodin (ALE)	0.2	0.008	60	0.2–2.0	0.9922	Linear	ALE-D4
Chrysophanol (CHR)	0.1	0.004	82	0.1–1.0	0.9920	Linear	ALE-D4
Danthron (DAN)	0.2	0.008	114	0.2–2.0	0.9931	Linear	DAN-D4
Emodin (EMO)	0.025	0.001	85	0.025–0.25	0.9942	Linear	EMO-D4
Frangulin A (FRA)	0.1	0.004	70	0.1–1.0	0.9920	Linear	ALE-D4
Frangulin B (FRB)	0.1	0.004	72	0.1–1.0	0.9921	Linear	ALE-D4
Glucofrangulin A (GFA)	0.1	0.004	33	0.1–1.0	0.9969	Linear	None
Glucofrangulin B (GFB)	1.0	0.042	42	1–10	0.9962	Linear	None
Physcion (PHY)	0.1	0.004	58	0.1–1.0	0.9951	Linear	None
Rhein (RHE)	0.1	0.004	87	0.1–1.0	0.9958	Linear	EMO-D4
Sennoside A (SENA)	1.0	0.042	47	1–10	0.9939	Linear	None
Sennoside A1 (SENA1)	0.75	0.031	51	0.75–7.5	0.9930	Linear	None
Sennoside B (SENB)	0.75	0.031	47	0.75–7.5	0.9920	Linear	None
Sennoside C (SENC)	0.5	0.021	42	0.5–5.0	0.9905	Linear	None

## Data Availability

The original contributions presented in the study are included in the article/supplementary material, further inquiries can be directed to the corresponding author.

## Supplementary Materials

The following supporting information can be downloaded at: https://www.mdpi.com/article/10.3390/toxins16120505/s1, Table S1: Overview of commercial samples purchased for the analysis of HADs, Table S2: Concentrations of HADs in commercial food supplements and herbal infusions, Figure S1: Influence of the different mobile phase modifiers and additives on the MS signal intensity of HADs. Figure S2: Efficiency (%) of the different solvents tested for the extraction of HADs from food supplements, Figure S3: LC-MS/MS multiple reaction monitoring (MRM) chromatograms of neat solvent MeOH/H_2_O (50/50, *v/v*); (lower chromatogram), blank extract of food supplement (middle chromatogram) and blank extract of food supplement spiked with individual HADs at levels corresponding to 0.075 mg kg^−1^ EMO, 0.15 mg kg^−1^ ALA, 0.3 mg kg^−1^ ALB, CHR, GFA, FRA, FRB, PHY and RHE, 0.6 mg kg^−1^ ALE and DAN, 1.5 mg kg^−1^ SENC, 2.25 mg kg^−1^ SENA1 and SENB, and 3 mg kg^−1^ GFA and SENA in a sample (upper chromatogram).
