# Supplementary material for: Determination of 16 Hydroxyanthracene Derivatives in Food Supplements Using LC-MS/MS: Method Development and Application
Source: Toxins (Basel). 2024 Nov 23;16(12):505. doi: 10.3390/toxins16120505 (PMC11728519; doi:10.3390/toxins16120505)
Supplement: Supplementary file 1 [file toxins-16-00505-s001.zip › toxins-3280959-supplementary.pdf]

Determination of 16 Hydroxyanthracene Derivatives in Food Supplements using LC-MS/MS: Method Development and Application

Supplementary material

Table S1. Overview of commercial samples purchased for the analysis of HADs.

Sample code	Product type	Country of production	Brief description of ingredients	Serving / Recommended intake
**S1**	Herbal infusion (dry)	Not specified	Apple, hibiscus, rosehip (10%), rhubarb (2.8%), rose buds, rose petals	1 ½ teaspoon per serving
**S2**	Herbal infusion (dry)	The Netherlands	Peppermint (52%), senna leaves (45%), goldenrod (1%), hibiscus (1%), lovage root	1 tea bag (1.5 g) per serving
**S3**	Food supplement	Belgium	Chinese rhubarb, dry extract of caraway (*Carum carvi* L.) 50 mg (per tablet), guar powder (*Cyamopsis tetragonoloba* (L.) Taub.) 18.75 mg, tamarind dry extract (*Tamarindus indica* L.) 5 mg, fig powder (*Ficus carica* L.) 5 mg	1 tablet a day
**S4**	Food supplement	Belgium	Fiber-rich powder of gum arabica*, hemp seeds (THC-free)*, grated coconut*, prickly pear cactus*, baobab fruit pulp*, psyllium husks*	Not specified
**S5**	Food supplement	Belgium	Chlorella* powder (*Chlorella pyrenoidosa*) 50%, spirulina* powder (*Spirulina platensis* / *maxima*) 50%	Not specified
**S6**	Food supplement	Italy	Senna powder (*Cassia angustifolia* M. Vahl.) leaves 249 mg (per tablet), dry extract of marshmallow (*Althaea officinalis* L.) 45 mg, Chinese rhubarb (*Rheum officinale* Baill.) rhizome 3 mg, Chinese cinnamon powder (*Cinnamomum zeylanicum* Nees.) bark 2.4 mg, clove powder (*Syzygium spiceum* (L.) Merril e LM Perry) 0.3 mg, propolis dry extract 3%	1 tablet a day

**S7**	Food supplement	Belgium	Brown rice protein powder*, powder from: acerola*, pomegranate*, blueberry*, spirulina*, sea buckthorn*, tomato*, matcha*, moringa*, kale*, nettle*, beetroot*, rosehip*, turmeric*, gotu cola*, *Aloe vera**	5-10 g per serving
**S8**	Food supplement	Belgium	Black psyllium powder (*Plantago afra*, seed) 18 mg (per 2 capsules), dandelion powder (*Taraxacum officinale* Weber, leaf) 16 mg, burdock powder (*Arctium lappa* L, root) 16 mg, fennel powder (*Foeniculum vulgare* Mill, fruit) 14 mg, fumitory powder (*Fumaria officinalis* L, top) 14 mg, artichoke powder (*Cynara scolymus* L, leaf) 10 mg, turmeric powder (*Curcuma longa*, rhizome) 10 mg, *Lactobacillus bulgaricus*, *Lactobacillus acidophilus*, *Bifidobacterium lactis*, *Lactobacillus casei*, *Streptococcus thermophilus*	2 capsules a day
**S9**	Food supplement	Belgium	Senna powder (*Cassia angustifolia* Vahl.) 364 mg (per 2 tablets), dry extract of senna (*Cassia angustifolia* Vahl.) 43 mg	1-2 tablets a day
**S10**	Food supplement	France	Slippery elm bark powder (*Ulmus rubra* Muhl.) 200 mg (per tablet), steviol glycosides	1 tablet a day (max. 3 tablets a day)
**S11**	Food supplement	Belgium	Lactic acid bacteria, extracts of *Frangula alnus* (buckthorn) 120 mg (per capsule), *Trigonella foenum graecum* (fenugreek) 40 mg, *Linum Usitatilisatum* (linseed) 25 mg, *Aloe vera* (aloe) 5 mg	2 capsules 3 times a day
**S12**	Herbal infusion (dry)	Belgium	Black elderberry* (*Sambucus nigra*), senna* (*Cassia angustifolia* 20%), chamomile* (*Matricaria chamomilla* 20%), fennel* (*Foeniculum vulgare* 15%), rosehip* (*Rosa canina* 10%), licorice* (*Glycyrrhiza glabra*)	1-2 tea bags (0.6-1.2 g) a day
**S13**	Herbal infusion (dry)	Belgium	Apple pulp* 45%, senna leaves* 35%, hibiscus flowers* 15%	1 tea bag (0.7 g) per serving
**S14**	Herbal infusion (dry)	France	*Rhamnus frangula*	1 teaspoon per serving
**S15**	Food supplement	Belgium	Powder (100%) from wheat grass juice*, chlorella*, curcuma*, dandelion*, fennel*, yacon*, nettle*, milk thistle*, artichoke	Not specified

**S16**	Food supplement	Belgium	*Aloe vera* extract 200:1 (75 mg) (1 capsule is equal to 15 g pure *Aloe vera* juice)	1-3 capsules a day
**S17**	Food supplement	France	Oat fibers 27.7% (*Avena sativa* L.), plum paste 22% (*Prunus domestica* L.), rhubarb paste 20% (rhubarb 42% *Rheum* x *hybridum Murray*), sunflower oil, German chamomille (*Matricaria recutita* L.), extract green anise 1% (*Pimpinella anisum* L.)	adult: 1-2 cubes a day; children 6-12 years old: 1/2 cube a day
**S18**	Food supplement	The Netherlands	Silverweed extract 40 mg, buckthorn bark extract 40 mg, birch leave extract 40 mg, rhubarb root extract 30 mg, senna pods 30.2 mg (18 mg sennosides)	1 tablet a day
**S19**	Food supplement	Belgium	Dirt tree bark	Not specified
**S20**	Herbal powder	Turkey	Senna, anise, wild rose, wild dill, nettle, peppermint, yarrow, calluna leaf, chamomile	Not specified
**S21**	Herbal infusion (dry)	Macedonia	*Sennae* folium, *Frangulae* cortex, *Menthae piperitae* folium, chamomile flower, *Carvi* fructus, *Foeniculi* fructus, *Liquiritiae* radix, *Juniperi* fructus	2 tea bags (1.5 g) per serving
**S22**	Herbal infusion (dry)	Thailand	Senna leaves, senna pods, green tea leaves, *Garcinia atroviridis*	1 tea bag (2.4 g) per serving
**S23**	Herbal powder	Turkey	Fennel, anise, senna, green tea, hibiscus, coconut, flaxseed	Not specified
**S24**	Food supplement	USA	Burdock root, dandelion root, hawthorn berry, milk thistle seed, fenugreek seed, ginger root, mullein leaf, horsetail, juniper berry, senna leaf	Adult: 3 capsules a day; children: 1 capsule a day

*organic cultivation

Table S2. Concentrations of HADs in commercial food supplements and herbal infusions.

Sample code	Concentration (mg kg^{-1})															
	**ALA**	**ALB**	**ALE**	**CHR**	**DAN**	**EMO**	**FRA**	**FRB**	**GFA**	**GFB**	**PHY**[1]	**RHE**	**SENA**	**SENA1**	**SENB**	**SENC**
S1	<LOQ	<LOQ	<LOQ	<LOQ	<LOQ	**2.60**	<LOQ	<LOQ	<LOQ	<LOQ	0.36	<LOQ	<LOQ	<LOQ	<LOQ	<LOQ
S2	<LOQ	<LOQ	**57.11**	1.33	<LOQ	**13.42**	<LOQ	<LOQ	<LOQ	<LOQ	<LOQ	123.38	826.48	333.75	1054.77	312.50
S3	<LOQ	<LOQ	**45.83**	97.68	<LOQ	**30.79**	<LOQ	<LOQ	<LOQ	<LOQ	33.13	287.62	93.16	33.28	98.82	18.53
S4	<LOQ	<LOQ	<LOQ	<LOQ	<LOQ	<LOQ	<LOQ	<LOQ	<LOQ	<LOQ	<LOQ	<LOQ	<LOQ	<LOQ	<LOQ	<LOQ
S5	<LOQ	<LOQ	<LOQ	<LOQ	<LOQ	<LOQ	<LOQ	<LOQ	<LOQ	<LOQ	<LOQ	<LOQ	<LOQ	<LOQ	<LOQ	<LOQ
S6	<LOQ	<LOQ	**99.40**	13.20	<LOQ	**21.66**	<LOQ	<LOQ	1.08	<LOQ	2.44	327.79	2529.08	874.31	3148.30	728.18
S7	<LOQ	<LOQ	<LOQ	<LOQ	<LOQ	<LOQ	<LOQ	<LOQ	<LOQ	<LOQ	<LOQ	<LOQ	<LOQ	<LOQ	<LOQ	<LOQ
S8	<LOQ	<LOQ	<LOQ	<LOQ	<LOQ	<LOQ	<LOQ	<LOQ	<LOQ	<LOQ	<LOQ	<LOQ	<LOQ	<LOQ	<LOQ	<LOQ
S9	<LOQ	<LOQ	**39.36**	0.99	<LOQ	0.91	<LOQ	<LOQ	<LOQ	<LOQ	<LOQ	258.49	3407.33	679.03	3424.13	572.82
S10	<LOQ	<LOQ	<LOQ	<LOQ	<LOQ	<LOQ	<LOQ	<LOQ	<LOQ	<LOQ	<LOQ	0.13	1.38	<LOQ	1.88	<LOQ
S11	**188.82**[2]	**268.93**	**12.41**	20.18	<LOQ	**26.48**	382.86	191.05	3455.93	1032.50	19.58	2.46	<LOQ	<LOQ	<LOQ	<LOQ
S12	<LOQ	<LOQ	<LOQ	<LOQ	<LOQ	<LOQ	<LOQ	<LOQ	<LOQ	<LOQ	<LOQ	<LOQ	<LOQ	<LOQ	<LOQ	<LOQ
S13	<LOQ	<LOQ	**47.84**	0.89	<LOQ	**8.23**	<LOQ	<LOQ	<LOQ	<LOQ	<LOQ	108.84	1568.83	852.65	2703.45	516.04
S14	<LOQ	<LOQ	<LOQ	51.59	<LOQ	**168.49**	205.30	199.99	10,551.85	8868.36	42.92	<LOQ	<LOQ	<LOQ	<LOQ	<LOQ
S15	<LOQ	<LOQ	<LOQ	<LOQ	<LOQ	<LOQ	<LOQ	<LOQ	<LOQ	<LOQ	<LOQ	<LOQ	<LOQ	<LOQ	<LOQ	<LOQ
S16	<LOQ	<LOQ	<LOQ	<LOQ	<LOQ	<LOQ	<LOQ	<LOQ	<LOQ	<LOQ	<LOQ	<LOQ	<LOQ	<LOQ	<LOQ	<LOQ
S17	<LOQ	<LOQ	<LOQ	<LOQ	<LOQ	0,44	<LOQ	<LOQ	<LOQ	<LOQ	<LOQ	<LOQ	<LOQ	<LOQ	<LOQ	<LOQ

S18	<LOQ	<LOQ	**56.51**	146.80	<LOQ	**38.96**	417.65	165.48	6658.37	2464.84	85.55	786.57	9323.29	4650.66	15,390.39	868.57
S19	<LOQ	<LOQ	<LOQ	79.96	<LOQ	**414.93**	456.20	463.42	19,813.00	15,387.98	77.10	<LOQ	<LOQ	<LOQ	<LOQ	<LOQ
S20	**31.85**	**70.63**	**149.46**	30.00	<LOQ	**503.80**	<LOQ	<LOQ	<LOQ	<LOQ	21.00	193.99	1497.96	487.99	2246.76	338.97
S21	<LOQ	<LOQ	**40.73**	16.71	<LOQ	**67.84**	70.35	75.88	3208.32	2904.48	16.99	112.57	1375.33	414.05	1635.51	503.04
S22	<LOQ	<LOQ	**115.75**	3.94	<LOQ	**21.99**	<LOQ	<LOQ	<LOQ	<LOQ	<LOQ	184.53	3082.43	665.76	3095.48	985.62
S23	<LOQ	<LOQ	**91.22**	1.94	<LOQ	**25.79**	<LOQ	<LOQ	<LOQ	<LOQ	0.30	158.95	2012.30	1689.72	2349.79	716.51
S24	<LOQ	<LOQ	**55.13**	0.67	<LOQ	**13.49**	<LOQ	<LOQ	<LOQ	<LOQ	<LOQ	137.16	2501.42	1833.97	2501.42	1139.63

[1] Corrected for recovery.

[2] The values highlighted in bold exceeded the level of 1 ppm for ALE, EMO and the sum of ALA and ALB representing a concern for public health according to [35].

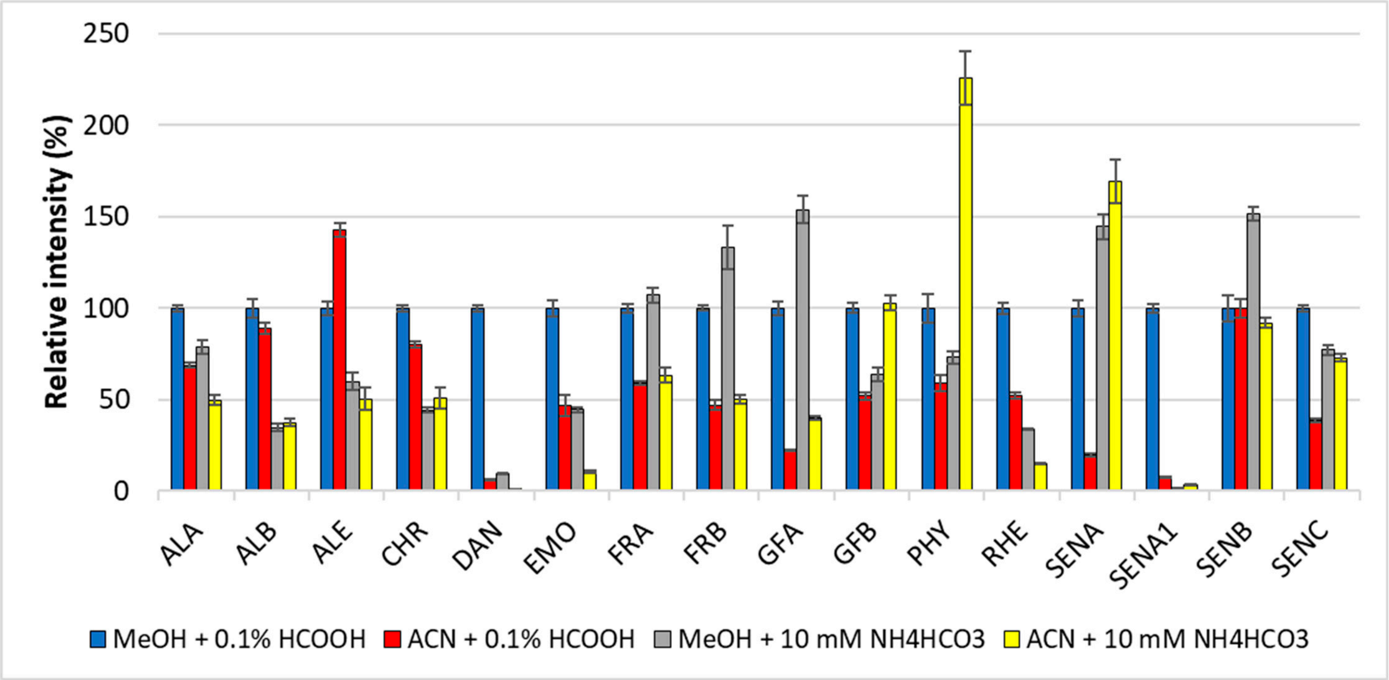

Figure S1. Influence of the different mobile phase modifiers and additives on the MS signal intensity of HADs. The error bars represent the standard deviation of the measurements. The relative intensity percentage was calculated for signal intensity in each mobile phase relative to the signal intensity recorded for mobile phase MeOH with 0.1% HCOOH.

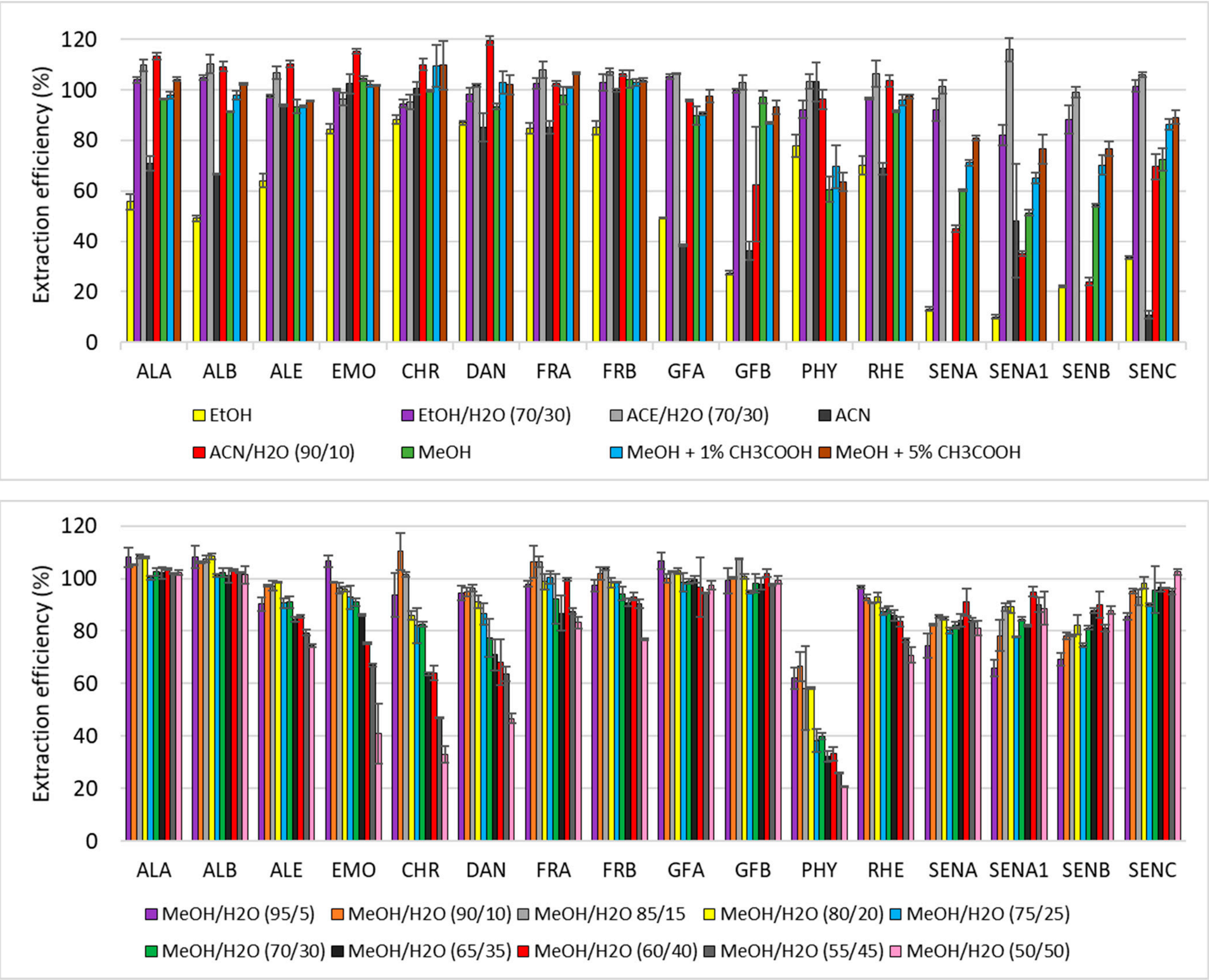

Figure S2. Efficiency (%) of the different solvents tested for the extraction of HADs from food supplements. For visibility reasons, the figure is split into two graphs. The error bars represent the standard deviation of the measurements.

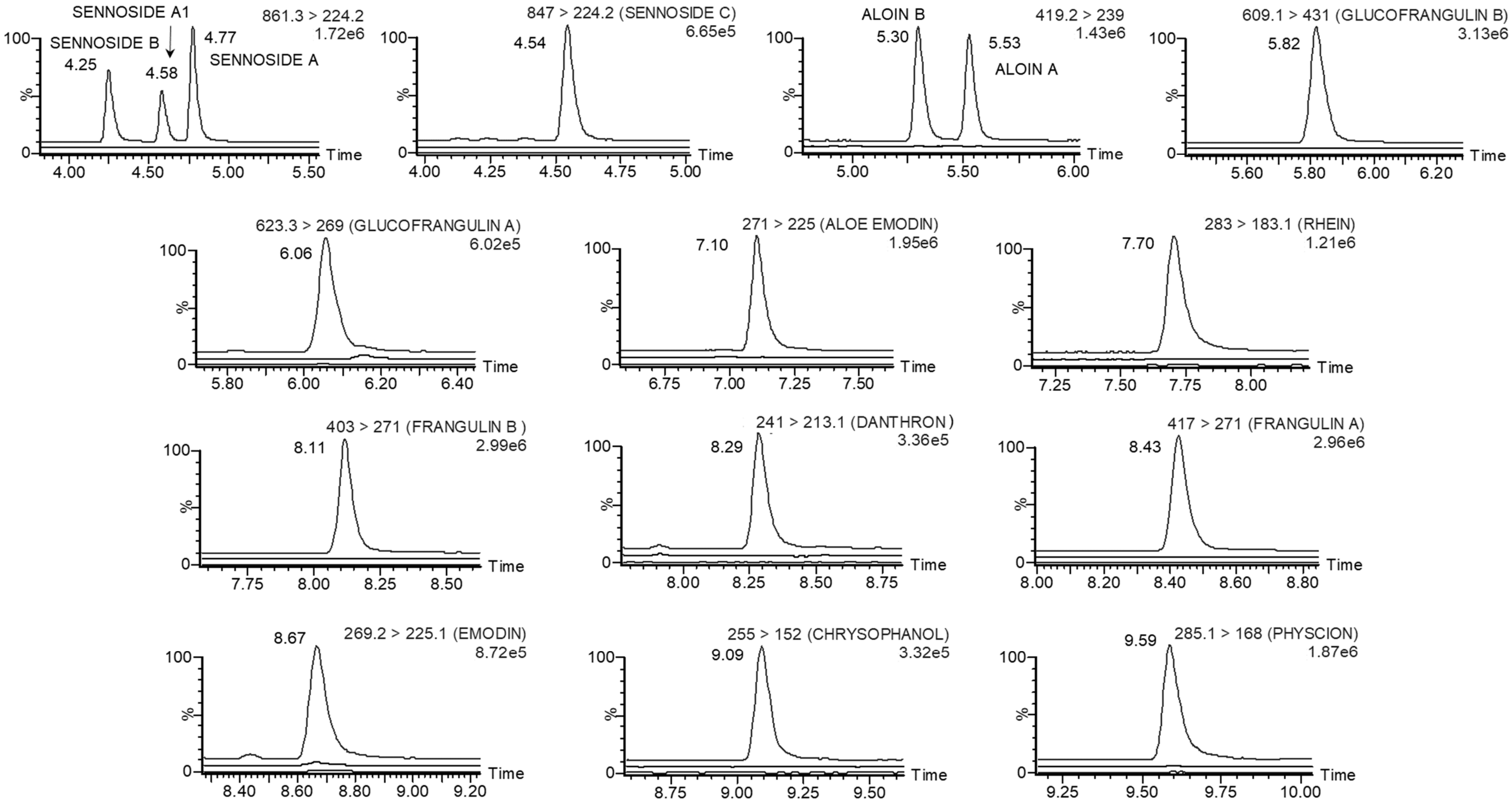

Figure S3. LC-MS/MS multiple reaction monitoring (MRM) chromatograms of neat solvent MeOH/H_2O (50/50, *v*/*v*) (lower chromatogram), blank extract of food supplement (middle chromatogram) and blank extract of food supplement spiked with individual HADs at levels corresponding to 0.075 mg kg^{-1} EMO, 0.15 mg kg^{-1} ALA, 0.3 mg kg^{-1} ALB, CHR, GFA, FRA, FRB, PHY and RHE, 0.6 mg kg^{-1} ALE and DAN, 1.5 mg kg^{-1} SENC, 2.25 mg kg^{-1} SENA1 and SENB, and 3 mg kg^{-1} GFA and SENA in a sample (upper chromatogram). For each analyte, the most abundant MRM transition is displayed. The vertical axes represent relative peak intensity (normalized to 100%), while the horizontal axes display retention time (in min). The chromatographic conditions used were as described in Section 4.4.
